# MetabOCT: a clinical trial looking for a metabolomic signature predicting the onset of Leber’s hereditary optic neuropathy in healthy MtDNA mutations carriers

**DOI:** 10.1007/s11306-025-02328-x

**Published:** 2025-09-09

**Authors:** Christophe Orssaud, Pascal Reynier

**Affiliations:** 1 Functional Unity of Ophthalmology, Ophthalmological Rare Diseases Center, ERN Eye, Georges Pompidou European Hospital, Paris, France; 2 Laboratoire de Biochimie et Biologie Moléculaire, Centre Hospitalier Universitaire, Angers, France; 3 Unité Mixte de Recherche (UMR) MITOVASC, Institut National de la Santé et de la Recherche Médicale (INSERM U1083), Centre National de la Recherche Scientifique (CNRS) 6015, Université d’Angers, Angers, France

**Keywords:** Clinical trial, Disease onset and progression, Leber hereditary optic neuropathy (LHON), Metabolomics, Retinal nerve fiber layer (RNFL)

## Abstract

**Introduction:**

The definition of Leber's hereditary optic neuropathy (LHON) does not take into account a preclinical phase during which the thickness of retinal nerve fiber layer (RNFL) is increased, prior to optic nerve atrophy, reducing the chances of visual recovery.

**Objectives:**

Search for a metabolomic signature characterizing this preclinical phase and identify biomarkers predicting the risk of LHON onset.

**Methods and results:**

The blood and tear metabolomic profiles of 90 asymptomatic LHON mutation carriers followed for one year will be explored as a function of RNFL thickness and compared to those of a healthy control.

**Conclusion:**

Identifying pre-clinical biomarkers would open a window for clinical trials.

## Introduction

Leber’s hereditary optic neuropathy (LHON, MIM#535000) is considered as the most common hereditary optic neuropathy (HON). It is caused by pathogenic variants in mitochondrial DNA (mtDNA), three of them being involved in more than 95% of cases, i.e. m.11778G > A in *MT-ND4* gene, m.3460G > A in the *MT-ND1* gene, and m.14484T > C in the *MT-ND6* genes. These mutations affect subunits of the NADH dehydrogenase, the complex I of respiratory chain (Carelli et al., 2017; Newman et al., 2020). The disease is maternally transmitted with an incomplete penetrance and a male predominance. Impairment of complex I activity notably cause increased production of reactive oxygen species and decreased ATP (adenosine triphosphate) production, leading to retinal ganglion cells (RGC) degeneration and apoptosis (Yu-Wai-Man et al., 2016; Newman et al., 2022). LHON prevalence is of about 1/50,000, with large variations according to the countries. However, it was recently shown that the frequency of LHON mutations observed in the general population was largely higher than expected given the prevalence of LHON (Watson et al., 2023). There are still some other unresolved question concerning this disorder such as the almost exclusive involvement of the optic nerves and the extremely brutal onset and progression of optic atrophy compared to other forms of HON. Clinically, LHON is actually characterized by a sequential, sudden and painless decrease of visual function occurring in both eyes in less than a month (Milea et al., 2010; Yu-Wai-Man et al., 2011). Visual impairment is severe, affecting the central visual field with peripheral vision preserved.

Many therapies have been tested but did not shown any therapeutic efficacy (Chen et al., 2022). There are currently two treatments which have shown therapeutic effects. Gene therapy with lenadogene nolparvovec (rAAV2/2-ND4) is the latest treatment tested with a moderate effect since mean visual acuities improvement for treated eyes were 1.23 logMAR (Biousse et al., 2021). Idebenone, an analog of coenzyme Q10, is the most commonly used treatment (Carelli et al., 2017; Klopstock et al., 2011). Although this molecule only slightly improves visual acuity, studies have reached a consensus to recommend its prescription within 18 months following the onset of visual acuity loss (Carelli et al., 2017), although improvements in visual function have been observed in some patients with a prescription beyond the second year (Pemp et al., 2021). But a potential deleterious effect of idebenone in patients carrying the m.3460G > A pathogenic variant was recently suspected (Yu-Wai-Man et al., 2024). Preventive therapy based on avoiding molecules toxic to mitochondria (i.e. alcohol and tobacco) and neurotoxic drugs is also recommended (Kogachi et al., 2019; Jurkute et al., 2019).

To explain the relative limited efficacy of treatments, it can be hypothesized that, as proposed by Newman, clinical trials are performed too late, at a stage at which the RGCs can no longer get back functional (Newman et al., 2005). A pre-clinical phase generally precedes the acute onset of visual acuity impairment, during which the optic disc is engulfed, and the thickness of retinal nerve fiber layer is increased on optical coherence tomography (OCT) examination (Carelli et al., 2017). This pre-clinical phase would appear an attractive favorable time to improve therapeutic efficacy in future clinical trials. It would be important to confirm this hypothesis of a favorable therapeutic window. The poor understanding of this initial phase of the disease means that its pathophysiology needs to be explored further, and that it would be useful to find more precise biomarkers of disease progression than visual acuity, visual field or OCT. These biomarkers may be able to detect the ocular anomalies, which herald an imminent onset of the disease in asymptomatic LHON mutation carriers. In this sense, metabolomics appears attractive since it allows a hypothesis free deep biological phenotyping with the potential to identify hitherto unexplored biomarkers.

That is the reason we initiated a prospective clinical trial performing a metabolomic study in blood and tears samples of LHON mutations asymptomatic carriers according to the thickness of their retinal nerve fiber layer in OCT.

## Protocol and testing

2.1. *Regulatory*: This clinical trial was recorded on Clinical Trial (NCT06682819) and approved by the Sud-Est I Committee for the Protection of Persons (Number: 22.01849.000134). The blood and tear samples will be included and analyzed as part of the Biological Sample Collection “Neurogenetic Diseases” of the Angers University Hospital. This collection was approved by the Committee for the Protection of Persons CPP Ouest II, Angers, France (identification number: CPP CB 2014/02; declaration number: DC-2011-1467; authorization number: AC-2017-2993). All participants will provide a written informed consent.
2.2. *Aim of the study*: The primary objective will be to determine the plasma and tears metabolomic profiles of healthy carriers of one of the three most frequent LHON pathogenic variants (m.11778G > A, m.3460G > A, and m.14484T > C) and to correlate them to the thickness of their retinal nerve fiber layer measured by OCT. The aim will be to identify one or more metabolites whose concentrations are significantly altered when the thickness of the retinal nerve fiber layer is increased.

This study will also include different secondary objectives. The plasma and tears metabolomic profiles from these healthy patients carrying LHON mtDNA mutations will be also compared with those of a control population not carrying mtDNA mutation to search for metabolic profiles specific of LHON asymptomatic carriers. The vitamin B3 and B9 status will be also explored in these healthy carriers according to the thickness of their retinal nerve fiber layer and in comparison, with the control population without mtDNA mutation. Indeed, in a previous metabolomics study we highlighted a vitamin B3 component (nicotinamide) deficiency in patients with clinical LHON disease (Newman et al., 2005). These vitamin assays will enable us to see whether changes in vitamin B3 and B9 concentration play a role in the disease pre-clinical stage and onset.

2.3. *Population*: We will include 90 patients that will be followed for one year. Due to the difference in LHON prevalence between men and women, and to a highly significant sexual dimorphism observed in metabolomics, the primary and secondary objectives will be evaluated separately according to the gender of healthy carriers and controls.

Inclusion criteria require that the subjects in both groups (healthy carriers and controls from 18 to 60 years old) will have a normal visual acuity in both eyes and no past history of optic nerve disease. Healthy carriers must harbor m.11778G > A, m.3460G > A, or m.14484T > C mtDNA pathogenic variants and healthy control will not carry any mtDNA pathogenic variants. Participants will have to accept the investigations (OCT, blood test, tears test) included in the protocol. Logically, the existence of any other forms of optic neuropathies, including glaucoma, will constitute an exclusion criterion.

2.4. *Study data*: The parameters studied in this study will be the far best corrected visual acuity recorded with an ETDRS scale and the RNFL thickness in OCT (Spectralis HRA – OCT2; Heidelberg Engineering GmbH, Hamburg Germany) at the inclusion and at one year. Patients will be contacted at 6 months to know if there is some evolution of their vision. Since vision decrease in LHON is often severe in a short time, we will consider if an optic atrophy has occurred.

More than 1000 metabolites will be measured in the blood and tears of participants by mass spectrometry, using the MxP500 XL kit from Biocrates. This kit allows a standardized approach based on internal quality controls and calibration curves similar to those used in medical biology. This experimental approach was previously achieved by the team in symptomatic LHON patients and results have been published (Newman et al., 2005). In addition, we will measure the vitamin B3 and B9 blood levels subjects.

2.5. *Statistic data*: Using the standardized data of metabolite concentrations meeting the quality criteria, univariate and multivariate statistical studies will be carried out in an unsupervised and supervised manner, in order to determine whether a discriminating biological signature is associated with the clinical phenotype (RNFL thickness). If a discriminating signature is identified, the metabolites involved in this signature will be analyzed as candidate biomarkers that may be used to predict the onset of the disease.

To compare continuous variables statistical analyses will be performed using SPSS (IBM SPSS Statistics, version 29.0). The normal distribution of quantitative data will be tested with Shapiro–Wilk test. Since values of both eyes will be analyzed together, generalized estimating equations (GEE) will be used to account for inter-eye correlations. For other values, Student t-test was performed using a significant level at p-values less than 0.05.

## Discussion

The blood metabolomic signature of the two most frequent hereditary optic neuropathies was established (Bocca et al., 2018, 2021). In OPA1-related dominant optic atrophy patients, the metabolic profile showed an impairment of the purine metabolism, including significant differences in xanthine, hypoxanthine, and inosine concentrations. In addition, the metabolomic signature of these OPA1 patients was characterized by differences in urocanate, choline, phosphocholine, glycerate, 1-oleoyl-rac-glycerol, aspartate, glutamate, and cystine concentrations (Bocca et al., 2018). The metabolomic signature of patients who developed LHON exhibited an impairment in 13 metabolite concentrations, such as dietary metabolites (nicotinamide, taurine, choline, 1-methylhistidine and hippurate), mitochondrial energetic substrates (acetoacetate, glutamate and fumarate) and purine metabolism (inosine) (Bocca et al., 2021).

However, we do not know when these impairments in metabolite concentrations occurred and if they are related for the occurrence of the LHON or if they are only a secondary consequence of the disease. In addition, whether asymptomatic LHON mutation carriers with paraclinical abnormalities such as optic disc swallowing or an RNFL increase thickness on OCT have still such typical LHON metabolomic signature is unknown. We decided not to include LHON patients in this study since we have already published a specific metabolomics signature in affected patients (Bocca et al., 2021). This will enable us to determine whether the metabolomic profiles of healthy carriers overlap or diverge from those observed in symptomatic individuals.

This study will be conducted to search for a metabolic signature specific to the LHON asymptomatic and presymptomatic periods. Such signature could suggest potential new therapeutic targets. We hope to be able to identify a metabolomic signature characterizing patients who are healthy carriers of LHON mutations, and a specific metabolomic signature of patients who will present an increase in the thickness of their RNFL suggesting this pre-clinical phase, or even the onset of optic atrophy, during the year in which these patients will be followed. Furthermore, the creation of groups based on gender will help to avoid bias due to estrogens, the role of which in the difference in prevalence between men and women is mentioned.

If this is the case, the early onset of an abnormal metabolic profile would demonstrate that RCG suffering begins before visual acuity declines. It would imply that the LHON occurrence begins before the decline in visual acuity becomes noticeable. This is a hypothesis proposed by that Newman (Newman et al., 2005). In primary open-angle glaucoma visual field scotomas appeared and visual acuity decreased after the disappearance of RGCs as confirmed by OCT (Cheng et al., 2024). Lowering the intra ocular pressure does not allow to recover the visual function except if some RGCs are only non-functional and able to recover normal function (Shin et al., 2024). The mechanism could be evoked in LHON to explain the relative limited recoveries after treatments such as idebenone or gene therapy. Some RGCs may indeed be permanently impaired, which would explain limited recovery regardless of the trial. At an earlier stage of the disease, before the RGCs are permanently destroyed, there is probably a better capacity for RGCs to become functional again. Clinical and paraclinical data tend to validate this hypothesis. A swelling of the RNFL in patients carrying the mutation or during a “pre-clinical” stage have been described (Carelli et al., 2017; Barboni et al., 2012). This so-called “pre-clinical” stage preceded the decrease of the visual acuity (Carelli et al., 2017). Such a stage must correspond to suffering of the RGCs and thus a real first stage of the disease. Visual loss should then be considered as an evolution of an infra-clinic optic neuropathy or as a complication. This will open a new window of opportunity to begin treatments as soon as there are biomarkers of RGC suffering.

## In conclusion

MetabOCT clinical trial we will try to identify biomarkers, through deep metabolomics phenotyping, that would be associated with the pre-clinical phase of LHON onset. These biomarkers would be useful to predict the risk of disease onset in asymptomatic LHON mutations carriers. These biomarkers would also be very useful for monitoring future trials aimed at treating patients during the pre-symptomatic phase, which may be more favorable to therapeutic effects.

## Data Availability

No datasets were generated or analysed during the current study.
